# Editorial Strategy to Get a Scholarly Journal Indexed by Scopus

**DOI:** 10.31138/mjr.32.1.1

**Published:** 2021-03-31

**Authors:** Armen Yuri Gasparyan, George D. Kitas

**Affiliations:** 1 Departments of Rheumatology and Research and Development, Dudley Group NHS Foundation Trust (Teaching Trust of the University of Birmingham, UK), Russells Hall Hospital, Dudley, West Midlands, United Kingdom; 2 Centre for Epidemiology versus Arthritis, University of Manchester, Manchester, United Kingdom

**Keywords:** Scopus, periodicals as topic, bibliography as topic, publishing, quality control, rheumatology

Journal indexing by global bibliographic databases has numerous implications for research and development in the era of digitisation. Researchers and authors employ bibliographic searches to comprehensively and systematically analyse relevant articles in their research and reviews. They consult lists of indexed sources at multidisciplinary and specialist databases to target relevant journals offering global visibility and dissemination of their own work. Research and academic institutions are also concerned with bibliographic records of their affiliated faculty and strive to expand indexing of their own periodicals to achieve and maintain ranking in the global competition.

Improved understanding of the journal indexing and its implications for all stakeholders motivates authors and editors to draft, edit, and publish quality work.^1^ To better serve their contributors’ and readers’ interests, editors should better organise their work and regularly consult experts with interest in bibliographic databases. Professional advice on instructions for authors, publication ethics statements, internationalisation, and digitisation pave the way toward expanded indexing.^2^ Such advice is particularly helpful for prioritising editorial tasks and applying to bibliographic databases in an organised way, when certain indexing criteria are met.^3^ The sequence of journal applications to information aggregators, digital repositories, and bibliographic databases is critically important. Failure to realise the importance of planning relevant activities may result in 2 to 4-year application embargos imposed by databases. Initial archiving by PubMed Central and listing by information aggregators such as EBSCO often allows garnering citations and acquiring journal metrics required for the coverage by prestigious abstract and citation databases such as Scopus.^3^ Over the past decade, Scopus has gained immense importance because of its constantly improving functionality, expanded coverage of quality sources, and scrutinised selection and deselection criteria. Launched in 2004 as an Elsevier database, Scopus has succeeded in outperforming many other indexing systems, with 22,794 active titles and more than 78 million bibliographic records.^4^ Equipped with advanced analytical tools and integrated with the social-media aggregator Plum Analytics, Scopus offers enriched scientometric and altmetric data at the level of individual articles. Scopus searches allow mapping science dynamics across academic disciplines and synthesising new scientific evidence. Accordingly, experts advise its inclusion in search strategies of narrative and systematic reviews.^5,6^ To ensure independent and comprehensive evaluation of journal application, the Scopus Content Selection and Advisory Board was established in 2009. The Board chairs monitor the multi-step evaluation procedure that facilitates acceptance of trustworthy sources with established quality controls, digitisation, and recognition by the international community. Re-evaluation mechanisms are also in place to analyse and delist sources with skewed metrics, un-ethical publishing practices, and diminishing value for the scientific community. Unsurprisingly, academic ranking systems such as the Times Higher Education (THE) and QS World University Rankings currently process Scopus data,^7^ motivating academic institutions to set goals for improved performance on this highly reputable database. Scopus coverage immediately boosts a journal’s influence on other indexed sources’ citation metrics. As such, validating and formatting article reference lists is crucial for entering and maintaining in the database. The indexing itself facilitates comprehensive and transparent impact calculation and journal ranking in a relevant subject category. Scopus-based metrics are publicly available on the database and the SCImago Journal & Country Rank platforms. Since June 2020, Scopus displays improved CiteScore values that cover a 4-year indexing window and distinguish journals with fast citation growth.^8^ All newly indexed journals with a publication history of 1 year have their monthly updated CiteScore values.^9^ In 2018, annual CiteScore values were available for 23,350 journals.^9^

The SCImago Journal & Country Rank platform currently displays much-desired metrics of 64 rheumatology journals (https://www.scimagojr.com/journalrank.php?-category=2745&page=1&total_size=64), including 16 in the highest quartile of the SCImago Journal Rank (SJR) with the most prestigious citations. The top 3 journals in this category are *Annals of the Rheumatic Diseases* (SJR 6.142), *Arthritis and Rheumatology* (4.113), and *Nature Reviews Rheumatology* (3.390). These flagship journals publish evidence-based articles of importance to the global community of rheumatologists and allied health professionals. Each of these journals has a long publishing history reflected in their *h*-index values of 228, 305, and 126, respectively. Such impressive metrics may stimulate editors of all other rheumatology journals to improve their adherence to the best standards in the field, solicit more evidence-based items such as practice guidelines, clinical trial reports and cohort studies, and gain more influence by attracting numerous relevant citations and the attention of respective professional communities.

In October 2020, the Scopus Content Selection and Advisory Board accepted the *Mediterranean Journal of Rheumatology* (MJR) for indexing, recognising the value of the new addition to the Scopus database and motivating the editorial team to achieve new highs. The acceptance for coverage is indeed a great achievement for the Journal editorial team, the Greek Rheumatology Society and Professional Association of Rheumatologists, and for the many rheumatology societies endorsing the Journal’s activities. Over the past few years, MJR has consistently upgraded its instructions and publication ethics statement, instituted posts for a Research Integrity Officer and Independent Statistician, internationalised its Board, expanded cooperation with numerous rheumatology societies, integrated with CrossRef and received Digital Object Identifiers (DOI), joined the Directory of Open Access Journals (DOAJ), and got archived by PubMed Central.^10^ Also, it signed the San Francisco Declaration on Research Assessment (DORA) and established a Social Media Coordinator post to diversify impact evaluation and accelerate dissemination of rheumatology research in the Mediterranean region and far beyond.

The indexing status is yet another major milestone for the MJR. With this and previous achievements in mind, the Editorial Team, supported by numerous authors, reviewers, and readers, anticipates an increased flow of great submissions and publication of truly influential articles of interest to all stakeholders.
